# Treatment outcomes of HIV patients with hepatitis B and C virus co-infections in Southwest China: an observational cohort study

**DOI:** 10.1186/s40249-021-00921-5

**Published:** 2022-01-06

**Authors:** Jingya Jia, Qiuying Zhu, Luojia Deng, Guanghua Lan, Andrew Johnson, Huanhuan Chen, Zhiyong Shen, Jianjun Li, Hui Xing, Yuhua Ruan, Jing Li, Hui Lu, Sten H. Vermund, Jinhui Zhu, Han-Zhu Qian

**Affiliations:** 1 SJTU-Yale Joint Center for Biostatistics and Data Science, Shanghai Jiao Tong University, Shanghai, China; 2 Department of Bioinformatics and Biostatistics, School of Life Sciences and Biotechnology, Shanghai Jiao Tong University, Shanghai, China; 3 Guangxi Key Laboratory of Major Infectious Disease Prevention Control and Biosafety Emergency Response, Guangxi Center for Disease Control and Prevention, Nanning, China; 4 Groton School, Groton, MA USA; 5 State Key Laboratory of Infectious Disease Prevention and Control (SKLID), Chinese Center for Disease Control and Prevention (China CDC), Collaborative Innovation Center for Diagnosis and Treatment of Infectious Diseases, Beijing, China; 6 School of Public Health, Yale University, New Haven, CT USA

**Keywords:** Hepatitis C virus, Hepatitis B virus, HIV, Antiretroviral therapy, Mortality, Retrospective cohort

## Abstract

**Background:**

Antiretroviral therapy (ART) has reduced mortality among people living with HIV (PLWH) in China, but co-infections of hepatitis B virus (HBV) and hepatitis C virus (HCV) may individually or jointly reduce the effect of ART. This study aimed to evaluate the impacts of HBV/HCV coinfections on treatment drop-out and mortality among PLWH on ART.

**Methods:**

A retrospective cohort study analysis of 58 239 people living with HIV (PLWH) who initiated antiretroviral therapy (ART) during 2010–2018 was conducted in Guangxi Province, China. Data were from the observational database of the National Free Antiretroviral Treatment Program. Cox proportional hazard models were fitted to evaluate the effects of baseline infection of HBV or HCV or both on death and treatment attrition among PLWH.

**Results:**

Our study showed high prevalence of HBV (11.5%), HCV (6.6%) and HBV-HCV (1.5%) co-infections. The overall mortality rate and treatment attrition rate was 2.95 [95% confidence interval (*CI*) 2.88–3.02] and 5.92 (95% *CI* 5.82–6.01) per 100 person-years, respectively. Compared with HIV-only patients, HBV-co-infected patients had 42% higher mortality [adjusted hazard ratio (a*HR*) = 1.42; 95% *CI* 1.32–1.54], HCV-co-infected patients had 65% higher mortality (a*HR* = 1.65; 95% *CI* 1.47–1.86), and patients with both HCV and HBV co-infections had 123% higher mortality (a*HR* = 2.23; 95% *CI* 1.87–2.66).

**Conclusions:**

HBV and HCV coinfection may have an additive effect on increasing the risk of all-cause death among PLWH who are on ART. It is suggested that there is need for primary prevention and access to effective hepatitis treatment for PLWH.

**Graphical Abstract:**

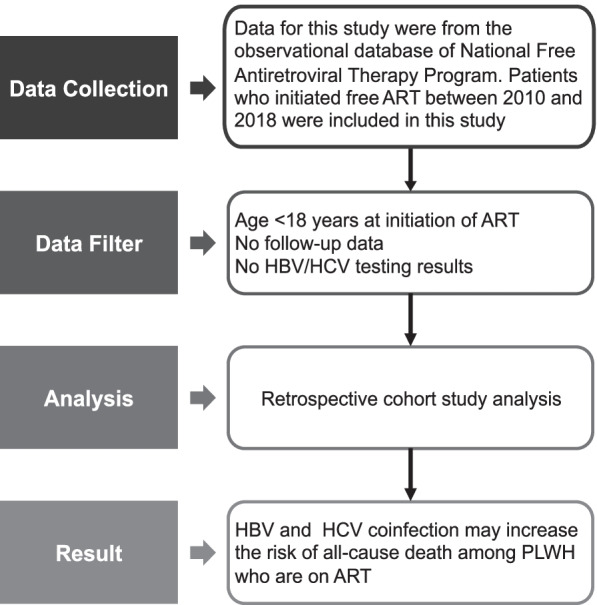

## Background

Highly active antiretroviral therapy (ART) has reduced deaths among people living with HIV (PLWH) in China and globally [1–3]. However, the effectiveness of ART depends on a variety of factors. Studies have shown that comorbid hepatitis B virus (HBV) and hepatitis C virus (HCV) infections could have negative impacts on HIV treatment outcomes, but few studies have assessed their individual and joint effects simultaneously [4–6]. HBV infection may accelerate the development of AIDS with HBV X proteins upregulating HIV replication and transcription by synergizing with kappa B-like enhancers and T-cell activation signals [7, 8]. HBV or HCV coinfection is associated with a higher level of hepatic fibrosis, which may impact the liver’s detoxification function [6, 9–11]. Since some ART drugs have liver toxicity, coinfection of HBV and HCV is a significant risk factor for death in PLWH.

Hepatitis B is endemic in China. Meta analyses showed that the prevalence of HBV infection was around 7% [12] among the adult general population of China and was double (13.7%) among people living with HIV (PLWH) [13]. Additionally, the prevalence of anti-HCV antibodies was lower among the general population (0.9%) [14], but higher among PLWH (24.7%) [13]. Triple infection of HBV, HCV and HIV occurred in about 3.5% of the population [13].

Though coinfections of HBV and HCV are common among PLWH, data on their effects on HIV treatment outcomes in China are sparse. We performed a retrospective cohort study analysis to evaluate the impacts of HBV/HCV coinfections on treatment drop-out and mortality among PLWH on ART in southwestern China.

## Methods

### Study design and study participants

This study was designed as a retrospective cohort analysis of HIV treatment data in the Guangxi Zhuang Autonomous Region in southwest China. As of October 2020, Guangxi represented 9.3% of the total number of nationally reported HIV/AIDS cases, and this region has accumulated the third highest number of HIV cases reported in China. Sexual transmission accounted for more than 95% of reported cases in Guangxi.

The data were from the observational database of the National Free Antiretroviral Treatment Program (NFATP) of China. The study subjects were HIV patients who received free ART between 2010 and 2018 through NFATP. Physicians administering the ART at the local hospitals managed case report forms at the time of initiating ART and follow-up at 0.5, 1, 2 and 3 months, and every 3 months thereafter. The case report forms were uploaded into a web-based database hosted by Chinese Center for Disease Control (China CDC). Eligibility criteria for the subjects of this study were: (1) HIV patients who initiated free ART between 2010 and 2018; (2) at least 18 years old; (3) tested for HBV or HCV; (4) provided informed consent. The researchers in the Guangxi Province CDC have access to all records in the NFATP for patients who lived in Guangxi Province.

Chinese free ART eligibility criteria have gone through several phases: From 2008, PLWH with CD4 cell counts lower than 350 cells/mm^3^ were eligible for treatment; since 2014, the treatment threshold was CD4 counts below 500; and since 2016, China has provided free ART for all PLWH regardless of CD4 count. Currently, first-line regimens for free ART in China are tenofovir (TDF) or azidothymidine (AZT) + lamivudine (3TC) + efavirenz (EFV) or nevirapine (NVP). Second-line regimens are TDF + 3TC + EFV or lopinavir/ritonavir (LPV/r).

### Data collection

Information about HIV patients in the electronic database NFATP includes two parts: baseline data and follow-up data. Baseline data included demographics such as age, sex, marital status and clinical characteristics such as route of HIV transmission, CD4 count (cells/mm^3^) before ART, WHO clinic stage before ART, initial first-line ART regimen, current ART regimen and calendar year of ART initiation. Follow-up data included transferal to another clinic, cessation of ART, loss to follow-up, duration of ART, and survival status. HBV infection was tested by finding Hepatitis B surface antigens (HBsAg) and HCV infection was tested by finding antibodies of HCV.

### Statistical analysis

We conducted a prospective follow-up study analysis. Time zero was defined as the date of ART initiation, and data was censored on December 31, 2019. Outcome variables included death and ART attrition. Survival status was recorded as censored if patients were still alive or transferred to another clinic. Attrition was defined as cessation of ART and loss to follow-up. Loss to follow-up or withdrawal of ART was defined as missing visits more than 90 days after the last record in a clinic. Incidence rates of mortality and attrition were calculated based on Poisson distribution and reported as the number of deaths and attritions per 100 person-years, respectively.

Cox proportional hazard models were used to evaluate the effects of baseline infection of HBV or HCV or both on death and attrition among PLWH. Competing risks for cause-specific hazard models were censored accordingly [20, 21]. Potential confounders were controlled by adjusting the model with the following baseline covariates: age, sex, marital status, route of HIV transmission, baseline CD4 count, WHO clinical stage before ART, initial first-line regimen, current ART regimen, duration of tenofovir disoproxil fumarate (TDF)-containing ART regimens, and calendar year of ART initiation.

Statistical significance was determined to have a two-sided *P* ≤ 0.05. All the statistical analyses were performed using SAS V9.1 (SAS Institute Inc., Cary, NC, USA).

## Results

### Baseline characteristics of study patients

**Fig. 1:**
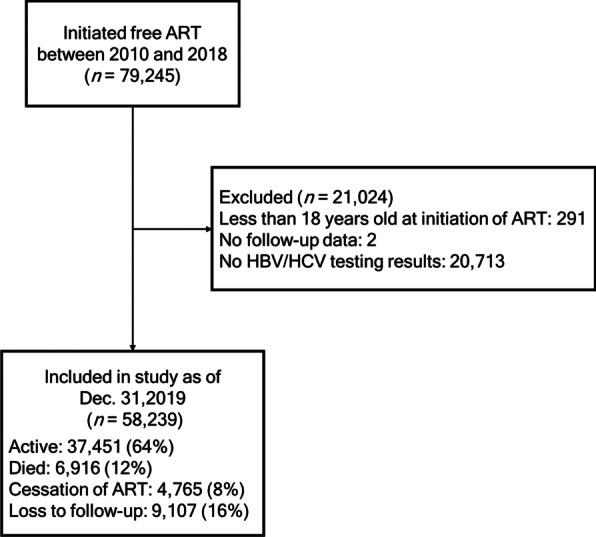
Flow chart of study sample selection. *ART* Antiretroviral therapy

As of December 31, 2019, 79,245 PLWH initiated free ART between 2010 and 2018 in Guangxi, China. Excluding 291 patients under 18 years old, two without follow-up data and 20,713 without HBV and HCV testing results, a total of 58,239 individuals were eligible and included in the analysis (Fig. 1). Of those included participants, 12% died, 16% were lost to follow-up, 8% dropped out of treatment and 64% were active on treatment by the end of follow-up.

The baseline characteristics of the study patients are shown in Table 1; 6,707 (11.5%) participants had HIV-HBV co-infection, 3,828 (6.6%) had HIV-HCV co-infection, 857 (1.5%) and had triple infection. Two fifths (40.9%) of patients were over 50 years old; 68.3% were male and 63.7% were married. The majority (87.3%) of patients were infected through heterosexual intercourse, followed by homosexual intercourse (5.9%), intravenous drug use (4.9%) and other causes (1.9%). Prior to ART initiation, 59.8% of the patients had CD4 counts ≤ 350 cells/mm^3^, and 5.9% of the patients were classified as WHO clinical stage III or IV. Patients with initial ART regimens of stavudine (D4T)-based, azidothymidine (AZT)-based, tenofovir disoproxil fumarate (TDF)-based and lopinavir-ritonavir (LPV/r)-based accounted for 8.5%, 33.7%, 46.8% and 10.1% of all patients, respectively. Most patients (78.7%) used first-line ART regimens, 21.3% used second-line regimens, and 43.1% used TDF-based ART regimens for more than 2 years.

**Table 1:** Baseline characteristics of HIV patients who initiated ART between 2010 and 2018 in Guangxi, China

Variable	Total	%	HIV only	%	HIV-HBV co-infection	%	HIV-HCV co-infection	%	HIV-HBV-HCV Triple infection	%
Total	58,239	100.0	46,847	100	6,707	100.0	3,828	100.0	857	100.0
Age, years										
18–50	34,424	59.1	25,731	54.9	4,504	67.2	3,404	88.9	785	91.6
≥ 50	23,815	40.9	21,116	45.1	2,203	32.8	424	11.1	72	8.4
Sex										
Male	39,754	68.3	31,097	66.4	4,798	71.5	3,110	81.2	749	87.4
Female	18,485	31.7	15,750	33.6	1,909	28.5	718	18.8	108	12.6
Marital status										
Married	37,104	63.7	30,197	64.5	4,306	64.2	2,136	55.8	465	54.3
Other	21,135	36.3	16,650	35.5	2,401	35.8	1,692	44.2	392	45.7
Route of HIV transmission										
Heterosexual intercourse	50,836	87.3	42,911	91.6	6,118	91.2	1,480	38.7	327	38.2
Homosexual intercourse	3,455	5.9	534	1.1	152	2.3	2,257	59.0	512	59.7
Intravenous drug use	2,827	4.9	2,488	5.3	297	4.4	36	0.9	6	0.7
Other	1,121	1.9	914	2.0	140	2.1	55	1.4	12	1.4
CD4 count before ART, cells/mm^3^										
≤ 350	34,837	59.8	28,133	60.1	3,853	57.4	2,345	61.3	506	59.0
> 350	23,402	40.2	18,714	39.9	2,854	42.6	1,483	38.7	351	41.0
WHO clinical stage before ART	47,524	81.6	38,079	81.3	5,513	82.2	3,187	83.3	745	86.9
I/II	7,268	12.5	5,937	12.7	807	12.0	443	11.6	81	9.5
III/IV	3,447	5.9	2,831	6.0	387	5.8	198	5.2	31	3.6
Initial first-line ART regimen										
ART containing D4T	4,966	8.5	4,011	8.6	412	6.1	430	11.2	113	13.2
ART containing AZT	19,621	33.7	17,208	36.7	1,027	15.3	1,176	30.7	210	24.5
ART containing TDF	27,246	46.8	20,270	43.3	4,634	69.1	1,881	49.1	461	53.8
ART containing LPV/r	5,863	10.1	4,862	10.4	610	9.1	319	8.3	72	8.4
Other	543	0.9	496	1.1	24	0.4	22	0.6	1	0.1
Current ART regimen										
First-line ART	45,854	78.7	36,743	78.4	5,311	79.2	3,107	81.2	693	80.9
Second-line ART	12,385	21.3	10,104	21.6	1,396	20.8	721	18.8	164	19.1
Duration of TDF -containing regimens										
≤ 2 years	33,115	56.9	27,865	59.5	2,740	40.9	2,088	54.5	422	49.2
> 2 years	25,124	43.1	18,982	40.5	3,967	59.1	1,740	45.4	435	50.8
Calendar year of ART initiation										
2010	3,515	6.0	2,518	5.4	437	6.5	425	11.1	135	15.8
2011	4,982	8.6	3,839	8.2	604	9.0	414	10.8	125	14.6
2012	6,226	10.7	4,876	10.4	700	10.4	537	14.0	113	13.2
2013	6,384	11.0	5,057	10.8	738	11.0	487	12.7	102	11.9
2014	7,290	12.5	5,755	12.3	883	13.2	558	14.6	94	11.0
2015	8,016	13.8	6,524	13.9	954	14.2	450	11.8	88	10.3
2016	7,421	12.7	6,170	13.2	823	12.3	349	9.1	79	9.2
2017	7,164	12.3	5,954	12.7	830	12.4	315	8.2	65	7.6
2018	7,241	12.4	6,154	13.1	738	11.0	293	7.7	56	6.4

*ART*, Antiretroviral therapy; *AZT*, Zidovudine; *D4T*, Stavudine; *LPV/r*, Lopinavir-ritonavir; *TDF*, Tenofovir; *HBV*, Hepatitis B virus; *HCV*, Hepatitis C virus; *HIV*, Human immunodeficiency virus

### Impact of HBV and HCV co-infections on death among PLWH who initiated ART

The unadjusted and adjusted effects of HBV and HCV co-infections on death are shown in Table 2. Among 58,239 patients who initiated ART between 2010 and 2018, 6,916 deaths were observed, and the overall mortality rate was 2.95 per 100 person-years [95% confidence interval (*CI*) 2.88–3.02]. The crude mortality rate was 2.86% in HIV-only patients, 2.84% in HBV-coinfected, 3.89% in HCV-coinfected and 4.66% in HBV/HCV-coinfected HIV patients. Multivariate cox models showed that compared with patients with HIV infection only, HBV co-infected patients had a 42% higher risk of death [adjusted hazard ratio (a*HR*) = 1.42; 95% *CI* 1.32–1.54; *P* < 0.001); HCV co-infected patients had a 65% higher risk (a*HR* = 1.65; 95% *CI* 1.47–1.86; *P* < 0.001); and patients with both HBV and HCV coinfections had a 123% higher risk (a*HR* = 2.23; 95% *CI* 1.87–2.66; *P* < 0.001). The increase of death risk among patients with triple infection (123%) approximately equals to the sum of increases in death among PLWH with co-HBV (42%) and those with co-HCV (65%) infection. There is an additive interaction between HBV- and HCV-co-infection on mortality among PLWH.

**Table 2:** Effect of HBV and HCV co-infections on death among HIV patients who initiated ART between 2010 and 2018 in Guangxi, China

Coinfection	Number of HIV patients	Deaths	Person-years (PY)	Mortality rate per 100 person-years (95% *CI*)	*HR* (95% *CI*)	*P* value	a*HR*^a^ (95% *CI*)	*P* value
Total	58,239	6,916	234,421.19	2.95 (2.88–3.02)				
HIV only	46,847	5,366	187,680.8	2.86 (2.78–2.93)	Reference		Reference	
HIV + HBV	6,707	797	28,092.09	2.84 (2.65–3.03)	0.99 (0.92–1.07)	0.784	1.42 (1.32–1.54)	< 0.001
HIV + HCV	3,828	590	15,148.30	3.89 (3.59–4.20)	1.35 (1.24–1.47)	< 0.001	1.65 (1.47–1.86)	< 0.001
HIV + HBV + HCV	857	163	3,500.00	4.66 (3.96–5.35)	1.60 (1.37–1.87)	< 0.001	2.23 (1.87–2.66)	< 0.001

*CI*, Confidence interval; *HR*, Hazard ratio; a*HR*, Adjusted hazard ratio; *HBV*, Hepatitis B virus; *HCV*, Hepatitis C virus; *HIV*, Human immunodeficiency virus

^a^Adjusted for Age, gender, marital status, route of HIV transmission, CD4 count before ART, WHO clinical stage before ART, initial first-line ART regimen, current ART regimen, duration of using TDF-containing regimens, calendar year of ART initiation

### Impact of HBV and HCV co-infections on treatment attrition among PLWH who initiated ART

The unadjusted and adjusted effects of HBV and HCV co-infections on treatment attrition are presented in Table 3. Among 58,329 patients, 13,872 patients dropped out from the treatment including 9,107 patients lost to follow-up and 4,765 stopping ART. The overall drop-out rate was 5.92 (95% *CI* 5.82–6.01) per 100 person-years. The crude drop-out rate was 5.42% in HIV-only patients, 5.13% in HBV-coinfected, 12.03% in HCV-coinfected and 12.51% in HBV/HCV-coinfected HIV patients. Multivariate cox models showed that compared to HIV-only patients, HBV co-infected patients were 34% more likely to drop out of treatment (a*HR* = 1.34; 95% *CI* 1.27–1.42; *P* < 0.001); HCV co-infected patients had a 73% increased risk (a*HR* = 1.73; 95% *CI* 1.61–1.87; *P* < 0.001); patients with both HBV and HCV co-infections had a 107% increased risk (a*HR* = 2.07; 95% *CI* 1.85–2.31; *P* < 0.001). The increase of attrition risk among patients with triple infection (107%) equals to the sum of increases in treatment attrition among PLWH with co-HBV (34%) and those with co-HCV (73%) infection. There is an additive interaction between HBV- and HCV-co-infection on treatment attrition among PLWH.

**Table 3:** Effect of HBV and HCV co-infections on ART attrition among HIV patients who initiated ART between 2010 and 2018 in Guangxi, China

Variables	Number of HIV patients	Attritions	Person-years (PY)	Attrition rate per 100 person-years (95% *CI*)	*HR* (95% *CI*)	*P* value	a*HR*^a^ (95% *CI*)	*P* value
Total	58,239	13,872	234,421.19	5.92 (5.82–6.01)				
HIV	46,847	10,169	187,680.8	5.42 (5.32–5.52)	Reference		Reference	
HIV + HBV	6,707	1,442	28,092.09	5.13 (4.87–5.39)	0.95 (0.90–1.01)	0.074	1.34 (1.27–1.42)	< 0.001
HIV + HCV	3,828	1,823	15,148.30	12.03 (11.50–12.57)	2.22 (2.11–2.33)	< 0.001	1.73 (1.61–1.87)	< 0.001
HIV + HBV + HCV	857	438	3500.00	12.51 (11.37–13.66)	2.31 (2.10–2.54)	< 0.001	2.07 (1.85–2.31)	< 0.001

*CI*, Confidence interval; *HR*, Hazard ratio; a*HR*, Adjusted hazard ratio; *HBV*, Hepatitis B virus; *HCV*, Hepatitis C virus; *HIV*, Human immunodeficiency virus

^a^Adjusted for Age, gender, marital status, route of HIV transmission, CD4 count before ART, WHO clinical stage before ART, initial first-line ART regimen, current ART regimen, duration of using TDF-containing regimens, calendar year of ART initiation

## Discussion

Our study confirmed the previous study finding that the Chinese national free ART program has significantly reduced HIV related mortality in China [15, 16]. The overall mortality rate in our study sample who started ART between 2010 and 2018 in Guangxi, China, was as low as 2.95 per 100 person-years. HBV and HCV co-infection could independently increase mortality. This is consistent with findings among the Asia–Pacific PLWH population [17]. In addition, co-infection with both HCV and HBV had an additive effect on the risk of death among PLWH.

Studies have shown that in China there is a high prevalence of HCV infection among people who inject drugs [18, 19], and injection drug use (IDU) is associated with faster HIV disease progression and increased risk of death [20, 21]. IDU is unlikely to explain the association between HCV infection and risk of death in our study, as only 4.8% of our study sample were PWID and IDU was adjusted for in assessing the association. HCV may cause hepatic fibrosis and reduce liver detoxification function [9, 10], which reduces patients’ tolerance to side effects of ART drugs and therefore increases HIV treatment drop-out and increases mortality. Our study also showed that patients with HCV co-infection were more likely to have treatment attrition than those without any co-infection, and this might be one factor explaining for the increased risk of death among PLWHI with HCV-co-infection.

Our study has limitations. First, HCV status was assessed by antibody testing in this study, and a positive HCV antibody test might indicate past or current infection. However, most HCV infections could become chronic as there was virtually no treatment for HCV patients in China during the study period, a positive HCV antibody test is a good indicator of HCV infection status. The misclassification of HCV infection to non-infection might be possible among a small proportion of infected individuals might experience spontaneous clearance after acute infection, but it could lead to bias toward a reduced effect size of HCV infection on ART attrition or death. Second, we assessed all-cause mortality rather than HIV-related mortality. Other potential confounders such as alcohol use and tuberculosis coinfection were not assessed and adjusted in the analysis, and they may have impact on the mortality. In addition, accounting for only HIV-related mortality would probably result in more accurate data about the effectiveness of HIV treatment and the adverse impact of HBV/HCV co-infection. Third, our study sample did not include PWID who had not started ART. About two-thirds of diagnosed PLWH in China have used ART [22]. Our study findings among those on ART may not be extrapolated to the one-third of PLWH who were not on ART. In addition, the transmission mode of the participants was dominated by heterosexual intercourse in Guangxi (87.3%, Table 1), while about two thirds of cases were from heterosexual transmission up to the end of 2015 across China. The mode of HIV transmission may affect the relationship between HBV/HCV co-infection and HIV treatment outcome. However, our analyses were adjusted for HIV transmission route. Therefore, HIV transmission mode may not affect the generalizability of our study finding.

Studies have assessed the individual effects of HCV or HBV co-infection on HIV treatment outcomes, but few studies have ever assessed the joint effect. Our study provided important evidence on both individual and joint impacts of HCV and HBV co-infections on mortality among PLWH. Antiviral drugs such as Mavyret® (glecaprevir and pibrentasvir) have revolutionized the treatment of hepatitis C, and it can cure the disease. However, these drugs are not included in the Chinese free ART program, and most patients with HCV infection have no access to these new medications. Considering high prevalence of HCV and HBV co-infections and their adverse effect on HIV treatment, the national HIV free ART program should incorporate screening and treatment for HCV and HBV infections.

## Conclusions

This cohort study showed that both HBV and HCV coinfection was associated with higher mortality and treatment attrition among PLWH who were on ART. There is need for primary prevention and effective hepatitis treatment among PLWH.

## Data Availability

The datasets are available from the corresponding authors on reasonable request.

## Abbreviations

HBV: Hepatitis B virus
HCV: Hepatitis C virus
HIV: Human immunodeficiency virus
ART: Antiretroviral therapy
PLWH: People living with HIV
IDU: Injection drug use
WHO: World Health Organization
NFATP: The National Free Antiretroviral Treatment Program
a*HR*: Adjusted hazard ratio
*CI*: Confidence interval
